# Phenotype MicroArrays as a complementary tool to next generation sequencing for characterization of tree endophytes

**DOI:** 10.3389/fmicb.2015.01033

**Published:** 2015-09-28

**Authors:** Kathrin Blumenstein, David Macaya-Sanz, Juan A. Martín, Benedicte R. Albrectsen, Johanna Witzell

**Affiliations:** ^1^Southern Swedish Forest Research Centre, Swedish University of Agricultural Sciences, AlnarpSweden; ^2^Department of Natural Systems and Resources, School of Forest Engineers, Technical University of MadridMadrid, Spain; ^3^Department of Plant Physiology, Umeå Plant Science Centre, Umeå UniversityUmeå, Sweden; ^4^Department of Plant and Environmental Sciences, University of CopenhagenCopenhagen, Denmark; ^5^School of Forest Sciences, Faculty of Science and Forestry, University of Eastern Finland, JoensuuFinland

**Keywords:** fungal phenotype, nutrient utilization, fungus–fungus interactions, phenolic compounds, Biolog PM

## Abstract

There is an increasing need to calibrate microbial community profiles obtained through next generation sequencing (NGS) with relevant taxonomic identities of the microbes, and to further associate these identities with phenotypic attributes. Phenotype MicroArray (PM) techniques provide a semi-high throughput assay for characterization and monitoring the microbial cellular phenotypes. Here, we present detailed descriptions of two different PM protocols used in our recent studies on fungal endophytes of forest trees, and highlight the benefits and limitations of this technique. We found that the PM approach enables effective screening of substrate utilization by endophytes. However, the technical limitations are multifaceted and the interpretation of the PM data challenging. For the best result, we recommend that the growth conditions for the fungi are carefully standardized. In addition, rigorous replication and control strategies should be employed whether using pre-configured, commercial microwell-plates or in-house designed PM plates for targeted substrate analyses. With these precautions, the PM technique is a valuable tool to characterize the metabolic capabilities of individual endophyte isolates, or successional endophyte communities identified by NGS, allowing a functional interpretation of the taxonomic data. Thus, PM approaches can provide valuable complementary information for NGS studies of fungal endophytes in forest trees.

## Introduction

The increasing interest in endophyte communities of plants, including those of forest trees, is fueled by the apparent potential of the endophytes to shape and modulate the stress tolerance in host plants, directly by priming or elevating defence responses in the plant (Rodriguez and Redman, 2008; Eyles et al., 2010; Albrectsen and Witzell, 2012; Witzell et al., 2014) or indirectly through competition for substrates (Blumenstein et al., 2015). Moreover, endophytes are explored as a potent source of new solutions, based on metabolites and enzymes, for industrial, pharmaceutical, or agricultural purposes (Rodrigues et al., 2000; Schulz et al., 2002; Rančić et al., 2006; Gaur et al., 2010). Investigations of endophytes have traditionally been constrained by the difficulty of deciphering the global endophyte communities that are hidden inside the plants and capturing the target species to cultures for functional studies at organismal level. Recent methodological advances may solve some of these problems. For instance, improvements in standard isolation and culturing processes are feasible (Kaewkla and Franco, 2013), and the accumulating information from next generation sequencing (NGS) studies is likely to support discovery of new endophytic species (Toju et al., 2013), and provide information about their characters (Lim et al., 2010). However, while the genomic NGS analyses now provide powerful tools for global high throughput analyses of endophyte communities (Unterseher et al., 2012), they are still limited by the need of conducting a destructive extraction procedure that creates a snapshot of the point-in-time status in the samples (Greetham, 2014). There is also an increasing need to calibrate the NGS-community profiles with relevant taxonomic identities of the microbes, and to further associate these identities with the corresponding functional traits.

The functional traits of organisms are expressed as phenotypic attributes, jointly defined by the genome and the environment (cf. Houle et al., 2010). In fact, it is the phenotypic characters that make up the desired outcome of any selection process and also the key to understanding the biological complexity (Houle et al., 2010; Cabrera-Bosquet et al., 2012). Adequate and reliable phenotyping is thus a crucial task, if we want to utilize the functional traits of endophytes for practical applications. In general, modern strategies for high throughput phenotyping of organisms (phenomics) include application of computer vision, imaging of cell traits using visible light, NIR and fluorescent imaging technology and reporter gene expression (Houle et al., 2010), but effective application of these methods in studies of the functional traits of endophytes is not uncomplicated. The taxonomic and morphological diversity and the physiological versatility of fungal endophytes further complicate the acquirement of biologically relevant information about their phenotypes. For instance, a major challenge in studies of fungal phenotypes is the indeterminate growth of the fungi, with mycelium that forms colonies and is composed of hyphae as the basic units (Davidson, 1998; Falconer et al., 2005). Moreover, the analysis is complicated by the fact that the fungal hyphae may behave collectively rather than as isolated modules, reacting to the conditions across the whole colony (Falconer et al., 2005). In the case of endophytic fungi, these challenges are magnified further, because the endophytes thrive inside the tissues and are often reluctant to be cultured on artificial media. Consequently, any phenotyping of fungal isolates is usually done using low throughput morphological or physiological measurements that despite their great value are often cumbersome.

An emerging method that seems to have a realistic potential to provide at high throughput information about the phenotypes of microbial isolates is the Phenotype MicroArray (PM) technique. This technique relies on microtiter-plate-based substrate utilization assays (Bochner and Savageau, 1977; Bochner, 1989; Bochner et al., 2001). PM technique provides (semi-)high throughput assays for characterization and monitoring the cellular phenotypes of pure cultures or communities in an environmental sample (Borglin et al., 2012). The cellular responses, i.e., respiration or growth, can be monitored over a period of time, which makes it possible to capture some of the metabolic dynamics of the target cells (Bochner et al., 2001). The method thus allows construction of specific metabolic fingerprints that can be used for identification of microbes with desired traits, e.g., for industrial applications (Greetham, 2014). So far, however, the method has mainly been used in studies of bacteria. For example, Dong et al. (2010) applied PM for identification of bacterial strains with capacity for converting a novel precursor into an anti-cholesterol drug. Recently, however, the method has gained popularity also in studies of fungi, including endophytes (Atanasova and Druzhinina, 2010; Blumenstein et al., 2015). For example, PM technology has been used to optimize growth media for production of secondary metabolites by filamentous fungi (Singh, 2009).

The aim of this method paper is to illustrate how PM methods can be applied in studies on the ecology and utilization potential of forest tree endophytes, and validate the performance and reliability of these methods. In particular, we report experiences from two procedures, one where we used pre-configured, commercially available PM arrays to evaluate nutritional niches of endophytes and pathogens sharing the same host plant (Procedure I; part of the results modified from Blumenstein et al., 2015), and another where we used an in-house configured PM array to test the sensitivity of endophytes to a set of carbon sources and inhibitory substrates (phenolic compounds), which were interesting for our research questions but not available among the preconfigured, commercially available arrays (Procedure II). Finally, we discuss the benefits and limitations of the PM approach in studies on the ecological role of tree endophytes and their utilization in practical applications.

## Materials and Methods

### Procedure I: Utilization of Pre-configured Biolog PM Plates for Comparison of Nutritional Niches of Endophytes and Pathogens

#### Selection of the Fungi for the Studies

We employed PM technique in studies where the aim was to experimentally explore the competitive interactions between pathogens and endophytes that co-exist in time and space in trees. For this purpose, the carbon and nitrogen substrate utilization patterns of two pathogens (causal agents of Dutch elm disease, *Ophiostoma novo-ulmi* and *O. ulmi*) and four endophyte species (see below) were studied. Three isolates per each of the *Ophiostoma-*species were purchased from CBS-KNAW Fungal Biodiversity Center, Netherlands, or originated from the mycology library of Spanish elm breeding program (Solla et al., 2008). Three of the studied endophytic fungi were isolated earlier from elm trees (*Ulmus* sp.) (Martín et al., 2013). Two of them, *Monographella nivalis* var. *neglecta* (three isolates) and *Pyrenochaeta cava* (two isolates) have earlier shown chemical antagonism against the pathogenic *O. novo-ulmi*. They both inhabit elm bark and xylem where interspecific competition for the niche might occur (Martín et al., 2015). The third fungus, *Aureobasidium pullulans* (three isolates), was included in the tests as an example of a ubiquitous, “generalist” fungus, with potentially broad nutritional niche. In nitrogen utilization tests, also a common biocontrol fungus, *Trichoderma harzianum* (MB#340299, purchased from CBS -KNAW Fungal Biodiversity Center, Netherlands) was included for comparison. All fungi were cultivated on malt extract agar (MEA) and 26°C following the recommended protocol (Biolog Inc.). Occasional light exposure was not excluded during the experiment period.

#### Preparation of Inoculum

For preparation of homogenous inoculum, we developed the following protocol that is carried out under sterile conditions. In order to obtain pure fungal mass and to avoid any contamination of agar in the inoculation fluid (IF), fungi were cultivated on semi-permeable cellophane membrane on MEA. After incubation at 26°C (10–15 days depending on species’ individual growth rate), the fungal mass was lifted from the cellophane membrane with a cotton swab. Material from 2 to 5 agar plates per isolate was found to contain sufficient fungal material for the tests. The material was transferred into 2 mL Eppendorf vials and manually homogenized with a pestle together with 500 μL of Biolog FF-IF. When a thick suspension was obtained, it was poured over cotton wool on a metal sieve placed over a beaker. By adding 1–3 mL FF-IF, the material was flushed through the cotton. The longer fungal hyphae and bigger cell aggregates were collected into the cotton wool, and a dense, homogenous solution containing fungal spores and small aggregates of mycelial cells was collected underneath the sieve. The viability of the cells in the suspension immediately after the collection procedure was tested by spreading 200 μL aliquots of the cell stock suspension on MEA plates. The viability of the cells in the suspension immediately after the cell collection procedure was tested by spreading 200 μL aliquots of the suspension on MEA plates. After 3–4 days incubation, outgrowing mycelium was visually checked for development and purity.

Using a turbidimeter, the optical density of the inoculum was adjusted to 62% by adding small amounts from the cell suspension. Depending on the species, 400 μL^-1^ mL of cell stock suspension per 17 mL FF-IF was used. Then, following the protocol from the manufacturer, solutions of glucose, sodium sulfate, and potassium phosphate were added. The final inoculum was transferred into a sterile reservoir for multichannel pipettes, and 100 μL of suspension was pipetted into each PM array well. The suspension was added to plates on the same day than it was prepared.

In order to count the colony forming units (CFU) of the inoculum, an aliquot of 100 μL was pipetted into Petri dishes containing MEA, gently tilting the dish with sterile glass beads to evenly distribute the fluid on the agar surface. After 3–4 days incubation, the CFU was determined.

#### Pre-configured Biolog Phenotype MicroArrays

The commercially available, pre-configured Biolog Phenotype MicroArrays (Biolog Inc., Harvard, CA, USA) are composed of microtiter plates with one negative control well and 95 wells pre-filled with a nutrient source (e.g., C,N,P,S, amino acids) or substrates leading to inhibitory conditions (pH, NaCl, antibiotics) in a dried state. The substrate rehydrates after the target cell suspension, mixed with an IF at a standardized cell density, is inserted in each well. The IF provided by Biolog contains nutrients or chemicals (e.g., C, N, P, S, K, Na, Mg, Ca, Fe, amino acids, purines, pyrimidines, and vitamins) at sufficient levels to maintain cell viability. Through this combination (a nutrient source or an inhibitory compound and IF), unique culture conditions are created for the inoculated cells (Bochner, 2009).

The phenotypic response, i.e., how the cells respond to the conditions, is monitored by the change of color or turbidity in each well. The IF contains a tetrazolium salt, which is reduced by the action of dehydrogenases and reductases of the prokaryote and yeast cells, yielding a purple formazan dye. This color reaction is irreversible, and thus the more intensive the stronger the organism is able to catabolize the provided substrate in the well. In other words, a color reaction indicates that the inoculated cells are actively metabolizing a substrate in the well, while the lack of color change implies that the cells are not able to utilize the substrate. The rate and extent of color formation in each well can be monitored at 490 nm and recorded by the OMNILOG instrument (Bochner, 2003), a specialized instrumentation provided by Biolog. Kinetic response curves can be generated for each well and used for cellular phenotype comparisons. Alternatively, color change can be recorded spectrophotometrically (Atanasova and Druzhinina, 2010), or by visual observations (Bochner et al., 2001). While the color reaction is most convenient for bacteria, the growth response of filamentous fungi can be recorded as change in the optical density at 750 nm (OD750) (Tanzer et al., 2003; Druzhinina et al., 2006; Seidl et al., 2006). Measurements of growth can also be conducted at 590 nm (Blumenstein et al., 2015), which yield results that are comparable to 750 nm.

In our Procedure I, we examined the nutritional niche of endophytes and pathogens with the Biolog plates: PM1 and 2A, that represent 190 carbon sources (95 on each plate) (Blumenstein et al., 2015) and PM3B with 95 nitrogen sources. Three (carbon-source studies, Blumenstein et al., 2015) or two (nitrogen-source studies) replicate plates were used.

#### Measurements and Data-Analysis

Data for fungal activity was obtained through measurements of OD at 590 nm (PM1 and PM 2A, Blumenstein et al., 2015) or 750 nm (PM3B) using a spectrophotometer with microplate format compatibility (SPECTROstar Nano BMG Labtech) every 24 h for 10 days. The first reading (T = 0) was done at approximately 30 min after the plates were inoculated with the fungal IF. In the PM3A (nitrogen test), the content of six randomly chosen wells of all 30 plates was placed on MEA plates after 360 h when the final reading was done in order to control possible contaminations and vitality of cells. The purity of the developing mycelium was observed during the seven following days.

Differences in OD for specific substrates were tested using standard ANOVA analyses. Global differences in substrate use were compared by implementing multivariate statistics or by calculation of a niche overlap index (NOI) which compares the number of substrates used and the intensity by which they are used between two strains of fungi (Blumenstein et al., 2015). The competitiveness of the focal fungus against another fungus, or the effectiveness of a potential biocide chemical, may thus be evaluated (Blumenstein et al., 2015).

#### Examples of the Application Potential of Pre-configured PMs: Nutritional Niche Studies with Elm (Ulmus sp.) Endophytes and Pathogens

Pre-configured PM plates were used to examine whether the carbon-substrate utilization profiles of elm endophytes differ from those of the Dutch elm disease pathogen (Blumenstein et al., 2015). The basic hypothesis to be tested was that endophytes with good potential as biocontrol agents should be able to effectively compete with the pathogen for carbon, but that a successful pathogen might also be superior competitor for nutrients against endophytes. Here, we discuss part of the results from the earlier study by Blumenstein et al. (2015). In ongoing studies, we are applying preconfigures PMs to study the same aspects in competition for nitrogen substances and present here some of the findings from these studies.

### Procedure II: In-house Configured PM Array to Test the Sensitivity of Endophytes to a Set of Inhibitory Substrates

#### Experimental Aim and Selection of the Fungi for the Studies

In order to explore the role of fungal endophytes in the early stages of wood degradation, we employed a combination of NGS and PM approaches. Endophytic fungi were isolated from the wood (including phloem and xylem, but excluding external bark) of *Eucalyptus globulus* and *E. camadulensis* twigs, 1–2 cm in diameter, collected in 2012 and 2013 from different provinces across Spain (five sites in five provinces, three trees per site). The collection was done in spring and the twigs were transported to the laboratory and stored at 4°C. On the same day or the day after, the twigs were surface sterilized with subsequent immersions (30 s in 70% ethanol, 5 min in 4% bleach and 15 s in 70% ethanol), followed by 15 min drying at room temperature. Then, the twigs were peeled with a sterilized scalpel to remove the external bark and 1–2 mm thick slices were excised and placed on 90 mm Petri dishes (four explants per dish). We used five different culture media: MEA, potato dextrose agar, yeast extract agar, rose Bengal chloramphenicol agar, and eucalypt sapwood agar (10% w/v eucalypt sapwood, and 3% w/v agar). During the following two weeks, emerging colonies were transferred into new MEA dishes for preparation of inoculum (see below). By DNA sequencing of the ITS region and searching for matches in the GenBank database (NCBI, Bethesda MD, USA) through BLAST algorithm (Martín et al., 2015) we identified the most probable family of each strain. Fifteen eucalypt endophyte isolates were used for test this procedure, belonging fourteen to the phylum Ascomycota and the remaining one to the phylum Basidiomycota. The ascomycetes were two sordariomycetes (orders Hypocreales and Microascales), one incertae sedis and the rest dothideomycetes. These last belonged to the families Dothioraceae (four strains), Pleosporaceae (three strains), Phaeosphaeriaceae (order Pleosporales), Lophiostomataceae, Botryosphaeriaceae, and Davidiellacea. Additionally, we included in our study *Trichoderma* sp., *Pycnoporus sanguineus* and *Trametes* sp. isolates, commonly used as model fungi, from our mycology library. The species *P. sanguineus* and *Tramete*s sp. are basidiomycetes of the order Polyporales.

#### Preparation of Inoculum

The selected isolates were cultured in Petri dishes on an autoclaved cellophane sheet over MEA medium (darkness, 25°C). After a week, the fungal biomass was harvested by rubbing with a sterilized scalpel and transferring the fungal tissue into a sterile centrifuge tube (15 mL) with a known volume of sterile distilled water. Centrifuge tubes were weighed before and after introduction of the tissue to calculate the weight of the added biomass. Then, the content of the centrifuge tube was homogenized using a sterilized tissue grinder, first with a large clearance pestle and then with a small clearance one (~20 strokes with each). The homogenate was inserted back into the centrifuge tube and stored at 4°C until use. The concentration of fungal tissue in the suspension was calculated and the suspension was diluted to 1 g/L before pipetting into the PM plates.

#### In-house Configured PMs

Optical 96-well, round-bottom, sterile polystyrene plates (Deltalab, Barcelona, Spain) were used in the modified PM tests. Each well was first filled with 60 μL of liquid basal culture media (35 μL for inhibition tests; see below), composed by autoclaved Murashige and Skoog (MS) salts (1x; ref. n. 0926230; MP Biochemicals; Santa Ana, CA, USA), Biolog Redox Dye E (2x; ref. n. 74225; Biolog Inc., Hayward, CA, USA), and filtered 1-metoxy-5-methylphenazine methosulfate (1.5 mg/l; ref n. A3799; Applichem, Darmstadt, Germany). We prepared the plates by pouring into each well 50 μL of MS salt solution (2x; i.e., two times as concentrated as the standard recipe; 25 μL of MS salt solution (4x) and glucose 1 M of C atoms for inhibition tests; see below), 10 μL of dye mix, which contained Biolog Redox Dye E (20x; provided by the manufacturer at 100x; this reagent’s final concentration was 2x) and of 1-metoxy-5-methylphenazine methosulfate (15 mg/L). MS salts are normally used to plant tissue *in vitro* culture, thus we expected they would also be appropriated for endophyte fungi. Biolog Redox Dye E is recommended by the manufacturer for assays with fungi. It changes its color from transparent to violet when reduced, in a similar way than the classical tetrazolium dye. The mediator 1-methoxy-5-methylphenazine methosulfate enhances the change of color.

After adding these components, each well was supplemented with selected substances (see the details below). Combinations of two groups of substances were tested: carbon sources and inhibitors (phenolic compounds). For testing the effect of seven different carbon sources (cellobiose, galactose, glucose, sucrose, xylose, pectin, and starch) on the growth of the fungi, we supplemented the media with 20 μL of carbon source solution, to reach a final concentration 0.25 M of C atoms in each well. For testing the possible inhibitory effect on the fungi by 10 phenolic compounds that have been associated to tree resistance as metabolites or external treatments (Witzell and Martín, 2008; Martín et al., 2010): chlorogenic, tannic, and gallic acids; the simple phenolics o-cresol, carvacrol, thymol, and phenyl alcohol, and the flavonoids catechins, myricetin, and quercetin). We supplemented the media with 50 μL of inhibitor solutions (2x). Water-insoluble compounds could dissolved in 10% ethanol (v/v; for stock solution: 2x), 25% methanol (v/v; for the stock solution: 5x), or an alkaline solution (0.01 M NaOH for the stock solution: 5x). To neutralize the alkalinity of the latter media, 10 μL of 0.02 M HCl was added into the wells before inoculation. To test how these solvents affect fungal metabolism, solutions of glucose and sucrose with all these three solvents were prepared to control their possible effect (see Effect of additive solvents).

After addition of the test substances, water was added to fill the volume in each cell to 90 μL. Finally, from a suspension of 1 g/L of homogenized fungal biomass (see above), 10 μL was added into each well, making up a final concentration of 0.1 mg of fungal biomass per mL.

The thermotolerant solutions (carbon sources, tannic, salicylic, and gallic acids) were sterilized by autoclaving and the thermolabile or volatile substances and the substances dissolved in alcoholic solution (chlorogenic acid, flavonoids, and simple phenols) were filtered through disposable, sterile cellulose acetate syringe filters of 0.2 μm pore size. Water was always deionized and autoclaved prior to use. All the operations were done under axenic conditions in a laminar flow chamber.

The PM plates (a total of 30) were composed following four general principles. First, each combination of carbon source or secondary metabolite with a fungal strain was replicated in three separate wells. Second, with few justified exceptions (see Unintentional Chemical Interactions), all wells of a single plate had the same concentration of inhibitory substances, ethanol, or methanol, whenever present. Third, all the treatments included one negative control with the relevant conditions, but fungal inoculum substituted by water, and another one containing inoculum, but the carbon source/secondary metabolite substituted by water. The first was used to calculate the net absorbance (see below), while the second was used as a reference to compare between different endophytic strains. Fourth, all plates were cultivated in the dark at 25°C.

#### Measurements and Data-analysis

The following aspects that have relevance for the applicability of PMs in our studies on tree endophytes were evaluated from the in-house configurated PMs.

##### Stability/repeatability

To evaluate the stability and repeatability of the designed configuration, we repeated assays with carbon sources (cellobiose, galactose, glucose, sucrose, xylose, pectin, and starch) with a six months interval, using the freshly prepared inocula of five eucalypt endophyte strains and a model fungus (*Trichoderma sp.*).

##### Unintentional chemical interactions on PM plates – volatility and unexpected color changes in the medium

Our preliminary tests indicated that certain volatile metabolites might affect cells in neighboring wells in a plate where no such substance had been added. This unwanted effect was evaluated in a plate as described above, where the first three columns were supplemented with 1 g/L (final concentration) of the simple phenol *o*-cresol, while the rest of columns were not. All wells possessed glucose 0.25 M of carbon atoms. In each row one different fungal strain was inoculated, except the last one that was a negative control.

Preliminary tests also indicated that in some inhibitory compound tests the culture media unexpectedly changed color to orange (note that dye should change to violet) when in contact with certain inhibitory substances and certain strains. To explore this phenomenon, we performed a test to infer if this change of color could be because the strains used certain phenolic chemicals as carbon sources. We tested thirteen endophyte strains (selected from the Spanish tree endophyte collection) and the two Polyporales model fungi in media with chlorogenic acid, gallic acid, and tannic acid (1 g/L final concentration, solved in water), salicylic acid (0.02 g/L, in water) and catechins (1 g/L final concentration, solved in ethanol) with glucose. Absorbance was later measured at λ = 405 nm and λ = 630 nm.

##### Effect of additive solvents

Because some of the phenolic compounds had to be dissolved in solvents (see above), we wanted to test if these affected fungal activity. Thus, we tested the same set of strains as in the preceding assay, in four solvents (ethanol, methanol, NaOH+HCl as described above, and water) with added sugar in the form of either glucose or sucrose (0.25 M of carbon atoms). To the basal culture media (MS salts+dye mix) we supplemented with glucose and 5% ethanol or water or with sucrose (0.25 M of carbon atoms) and methanol 5%, NaOH+HCl 0.002 M (i.e., saline solution) or water. Growth in the media with ethanol, methanol, and saline solution were compared to the growth in water.

##### Data analysis

We defined and calculated the following parameters from the absorbance reads: (i) gross absorbance (*A*_λ_): the mean of the three technical replicates (same conditions in three different wells) for each substance tested, measured at a given λ; (ii) net absorbance (*A*′_λ_ = *A*_λ_-*A*_λneg_): the difference between the gross absorbance and its negative control (water instead of fungal inoculum, *A*_λneg_); (iii) cumulated growth (*A*^d^_λ_ = *A*′_λ_^d^–*A*′_λ_^0^): net absorbance at day d (*A*′_λ_^d^) minus net absorbance at day 0 (*A*′_λ_^0^); and (iv) relative growth (ρ_λ_): the ratio between the cumulated growth in two different substances (*A*^d^_λsubst1_ : *A*^d^_λsubst2_), usually one of them taken as reference (glucose or sucrose). Comparisons between different strains were done in terms of their relative growth, and therefore the negative control was not considered in comparisons. Standard analyses of Pearson correlation, one-factor ANOVA and Principal Component Analysis were carried out in STATISTICA V8.0 (StatSoft Inc., Tulsa, OK, USA).

#### Examples of the Application Potential of In-house Configured PMs: Chemical Sensitivity of Eucalypt (*Eucalyptus* sp.) Endophytes

Phenolic compounds have been identified as potential plant internal defenses and as external inducers of plant defenses (Witzell and Martín, 2008; Martín et al., 2010). However, little is still known about the possible responses of endophytes to these chemicals. Thus, the in-house configuration of PMs with phenolic compounds was designed to evaluate the role of these compounds for individual endophyte species, with the underlying hypothesis the compounds would show inhibitory effects on fungi, but that the effect would show strain- and compound-specificity. Specifically, we performed an inhibition test of four phenolic compounds (phenol, *o*-cresol, thymol, and carvacrol) and two flavonoids (quercetin and myricetin) on the same 15 strains tested above plus a negative control without inoculum. Final concentrations were 0.1 g/L for the phenols and 0.01 g/L for the flavonoids. Flavonoids and carvacrol were dissolved in ethanol, whereas the other phenolics were dissolved in water. We incorporated the results on this assay to the ones on the test we did to research on undesired changes of color (inhibitory substances: chlorogenic, salicylic, gallic and tannic acids, and catechins). We only took into account measures at λ = 630 nm to minimize interferences of undesired color changes.

Optical densities were measured in a microplate absorbance reader ELx808 (BIOTEK, Winooski, VT, USA). We measured at λ = 405, 490, and 630 nm, and at 0, 1, 2, 3, 4, 5, 7, 9, and 11 days after inoculation (dai). Wavelengths were selected in order to detect if the absorbance shifts were due to an increase in the turbidity, a change of color due to Biolog Redox Dye, or a change of color by other causes. Single measurement was considered sufficient, because variation between repeated, consecutive measurements were found to be negligible in preliminary tests (Macaya-Sanz, personal observation) Absorbance measurements were stored using the software KCjunior provided by the plate reader’s manufacturer. The plates were also photodocumented at 0, 5, and 11 dai.

The absorbance values at all the wavelengths, but especially at shorter ones, were due to increase of turbidity of the medium and the cumulative quantity of redox reactions (reflected in the change of color of the Redox Dye), i.e., two interrelated processes, and were thus considered a proxy of the catabolic activity and the vegetative growth of the fungi.

## Results

### Standardization of Inoculum (Procedure I and II)

Adequate quality inoculum for PM tests was achieved from the studied endophytes through both procedures. In Procedure I, the inoculum concentration was determined by transmittance, whereas in Procedure II, the inoculum was standardized by biomass. Standard culture conditions were used for studied fungi in both Procedures (I and II), resulting in adequate amount of viable fungal biomass.

In Procedure II studies, we found that fungal inocula lost vitality after a month storage at 4°C, showing clearly reduced growth rate (Macaya-Sanz, personal observation). All the inocula were, however, alive after the storage period.

**FIGURE 1:**
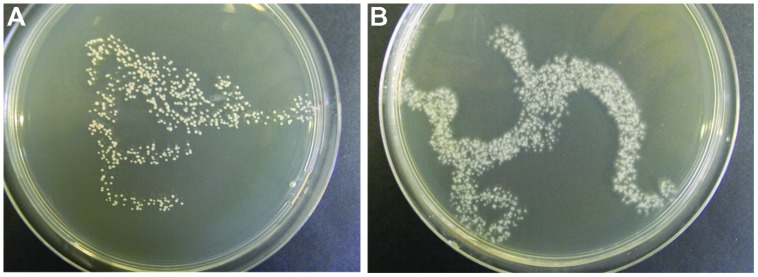
**Examples of fungal inoculum (100 μL, i.e., the volume injected into one well) applied to an agar plate for testing the development of CFU.** *Aureobasidium pullulans* **(A)** and *Ophiostoma ulmi* **(B)**.

In Procedure I, the test for the CFU in the inoculum gave varying results for the different species. For instance, CFU for *A. pullulans* was about 400 CFU per 100 μL (**Figure 1A**), whereas for *O. ulmi* the number of growing colonies was too dense to be counted (**Figure 1B**). Bacterial or fungal contaminations were not detected among the growth recovered from the randomly chosen wells.

### Technical Challenges with In-house Configurated PMs (Procedure II)

**FIGURE 2:**
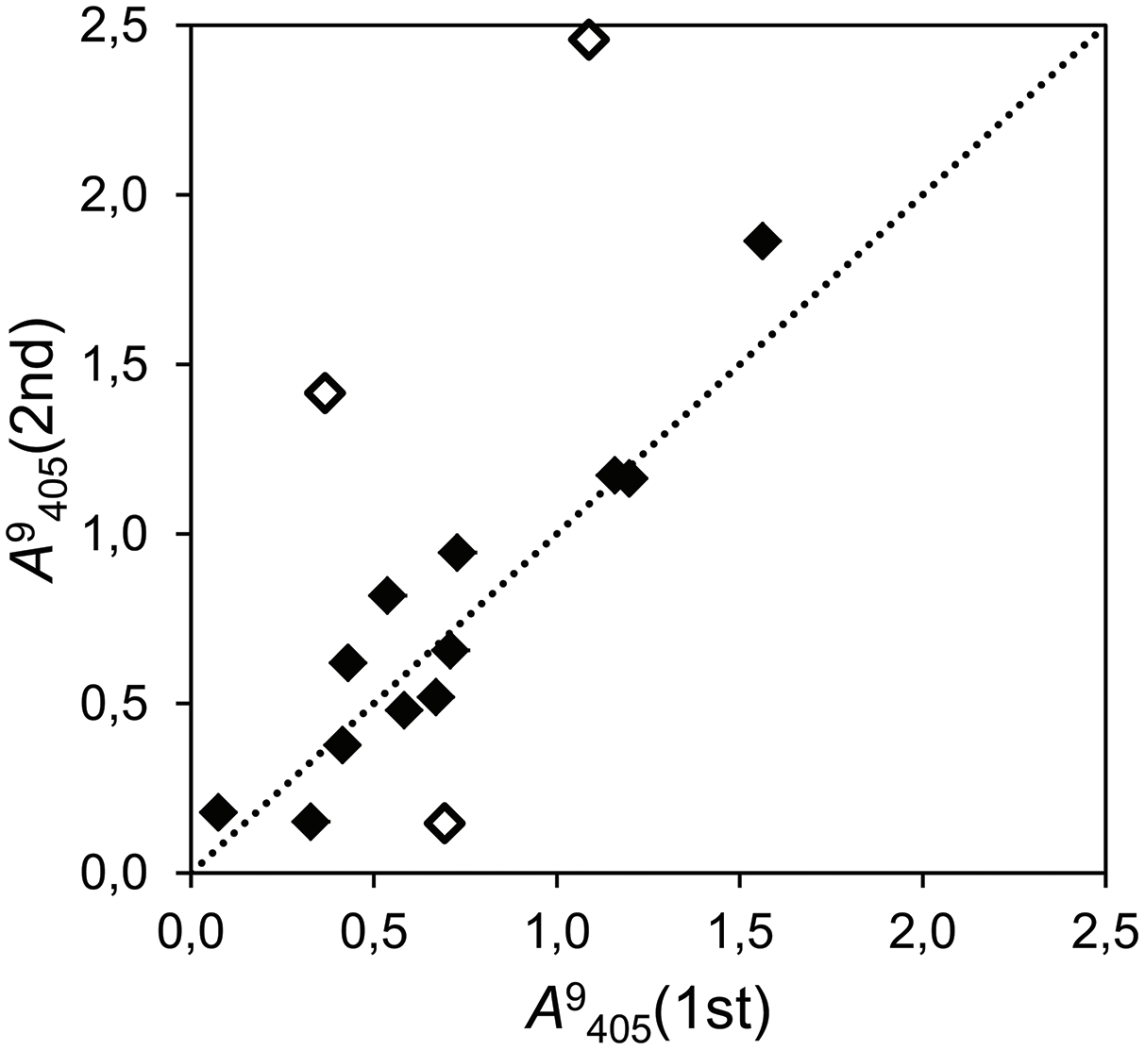
**Correlation plot of two independent assays of cumulative growth with glucose as the only carbon source (time point 9 days after inoculation (dai), λ = 405 nm) with 15 fungal strains, of which 13 were isolated as endophytes (*r*^2^ = 0.485; *P* = 0.004).** The dotted line represents the bisector of slope *b* = 1. Dots close to this line produced even results in both assays (filled dots). Empty dots indicate deviating strains.

The repeated assays showed that the precision of the in-house configurated PMs was moderate. In the experiment which tested 15 strains in glucose (**Figure 2**), the correlation was moderate (*r*^2^ = 0.485) but significant (*P* = 0.004), and the slope of the regression line was close to the unity (*b* = 1.17). Nevertheless, ignoring the results of three strongly deviating strains (marked with blank dots in **Figure 2**), the correlation grew to *r*^2^ = 0.889 and the regression slope shifted slightly toward one (*b* = 1.10).

The repeated assays where six strains were tested on seven carbohydrates, the precision (measured as correlation of cumulated growth) was extremely high (*r*^2^ > 0.9) in some of them, whereas almost negligible in others. Intriguingly, carbon sources where the standard deviation of the absorbance was low (i.e., the different response to the carbon source of the tested strains), displayed a reduced correlation. The carbon sources with high correlation also presented high standard deviation and values of linear regression slope close to the unity (**Table 1**). However, a couple of carbon sources did not follow this pattern (especially, xylose).

**Table 1:** Correlations between the growth of six fungal strains on seven different carbon sources at 9 days after inoculation (dai; λ = 405 nm) in two independent assays with identical conditions.

Carbon source	*A*^9^_405_(1)	*A*^9^_405_(2)	σ	*r*^2^	*P*	*b*
Cellobiose	1,258	0,830	0.433	0.179	0.403	0.5555
Galactose	0,747	0,849	0.165	0.002	0.940	–0.0361
Glucose	0,916	0,859	0.275	0.039	0.708	0.1372
Sucrose	0,646	0,658	0.381	0.986	0.0001	1.1845
Xylose	1,073	0,723	0.385	0.009	0.857	0.0681
Pectin	0,219	0,474	0.359	0.910	0.012	1.0413
Starch	0,860	0,341	0.441	0.977	0.001	0.6013

*The second and third columns display the mean of the cumulated growth of the six strains for the first and second assays, respectively. The fourth column shows the standard deviation (*σ*) of the pooled measurements from both assays. The fifth and sixth columns display the determination coefficient between two assays (*r*^*2*^) and its level of significance (*P*). The last column presents the slope of the regression line (*b*). Note that carbon sources with lower standard deviation have low correlations between assays. However, not all the carbon sources with higher standard deviation present high correlations.*

**FIGURE 3:**
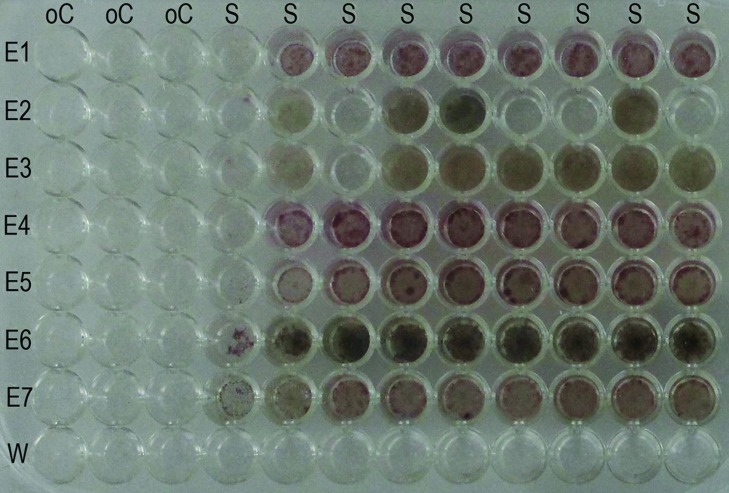
**Effect of volatile inhibitory substances on adjacent cells.** All the wells of the plate were supplemented with the standard basal medium. In each row, cell suspension of one endophyte strain was added, excluding the last row one where water was added. In the first three columns, the phenolic compound *o*-cresol was incorporated to a final concentration of 1 g/L. Note that the fungi in the fourth and fifth columns were partially inhibited in their growth. E = endophyte; W = water; S = standard basal medium; oC = standard basal medium supplemented with *o*-cresol.

Our tests with *o*-cresol indicate that there is a risk that the volatile compounds cause unintentional effects in the neighboring wells: we found that the fungal growth was severely reduced in the adjacent wells and visibly limited in the next columns (**Figure 3**).

**FIGURE 4:**
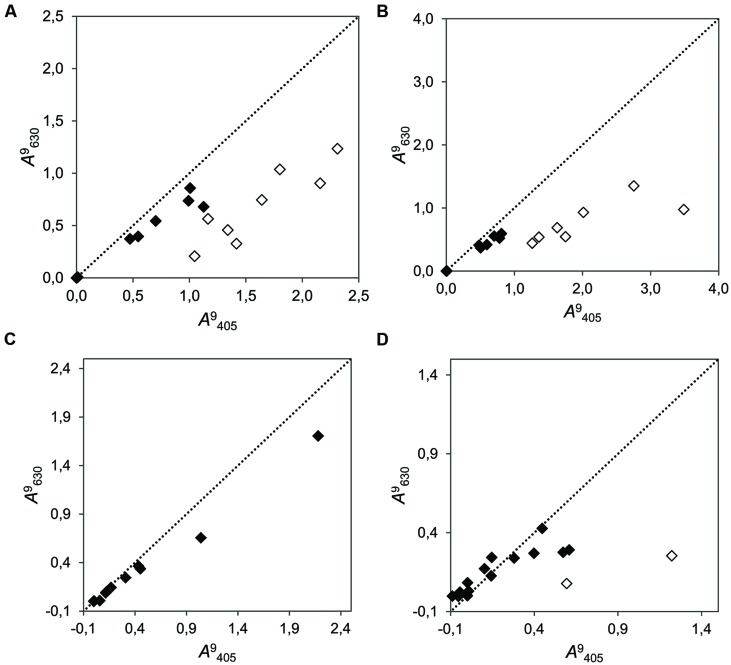
**Correlation plots of the cumulative growth (measured at λ = 405 nm vs. λ = 630 nm at time point 9 dai) of 15 fungi in media supplemented with four different phenolic compounds. (A)** Chlorogenic acid (*r*^2^ = 0.683; *b* = 0.430); **(B)** Gallic acid (*r*^2^ = 0.788; *b* = 0.321); **(C)** Salicylic acid (*r*^2^ = 0.992; *b* = 0.460); and **(D)** (+)-catechin (*r*^2^ = 0.407; *b* = 0.240). The dotted line represents the bisector of slope *b* = 1. Dots close to this line produced even results when measured with both wavelengths (filled dots). Empty dots indicate strains in which an unexpected change of color to yellow–orange was visually evident. Note that it is not expected that points aggregate to the bisector, because the measurements at different wavelengths need not to be alike, regardless of the undesired color change.

Our tests confirmed that unexpected color change (to orange) occurred only in certain combinations of strain and inhibitory substances. The combination of certain strains with the four tested secondary metabolites (salicylic acid, tannic acid, chlorogenic acid, gallic acid, and catechins) resulted in change of color to yellow–orange in last three of them. Occasional change of color was also found in tannic acid assays. This change of color was measurable as a shift in the ratio between absorbance at wavelength λ = 405 nm and at λ = 630 nm. In the cases were a change of color occurred, the absorbance at λ = 405 nm increased abnormally, and the ratio λ = 405 to λ = 630 was not conserved (**Figure 4**). Such color change did not occur when other strains were combined with these metabolites or when the strains were growing without these substrates.

**FIGURE 5:**
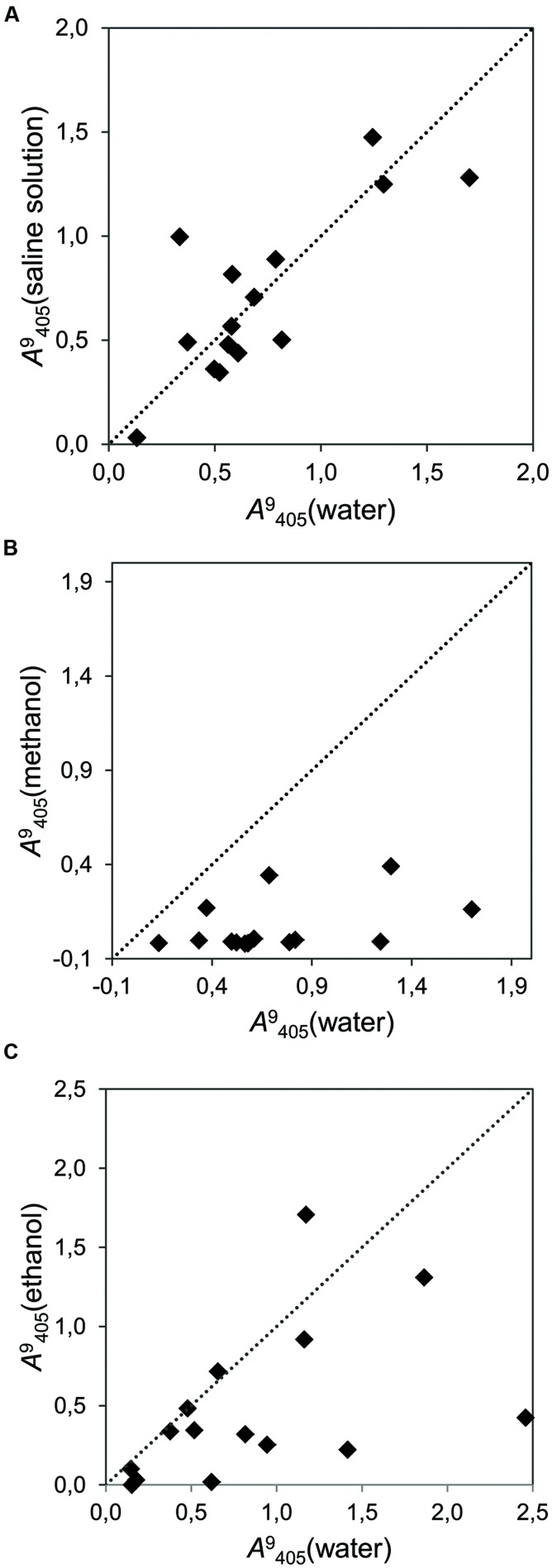
**Correlation plot reflecting the effects of different solvents on the cumulative fungal growth (time point 9 dai, λ = 405 nm) of 15 fungal strains.** Each dot represents one strain. The dotted line represents the bisector of slope *b* = 1. Placement of dots under this line indicates inhibition by the tested solvent. **(A)** Growth in a saline solvent (NaOH+HCl) versus water with sucrose as carbon source (*r*^2^ = 0.634); **(B)** Growth in methanol versus water with sucrose as carbon source (*r*^2^ = 0.178); **(C)** Growth in ethanol or water with glucose as carbon source (*r*^2^ = 0.230).

Tests with different solvents showed that some of them have a strong effect on the activity of the strains. Alkaline solution, which was neutralized with an acid, did not affect the activity of the strains (**Figure 5A**), while 5% methanol in water (v/v) induced a general decrease in the growth of all the endophytic strains (**Figure 5B**). Ethanol (5% in water, v/v) had an inconsistent effect on strains, decreasing the growth in some of them, but promoting it in others (**Figure 5C**).

### Application of PM to Forest Tree Endophyte Studies

#### Procedure I – Comparison of Nutrient Utilization Patterns

**FIGURE 6:**
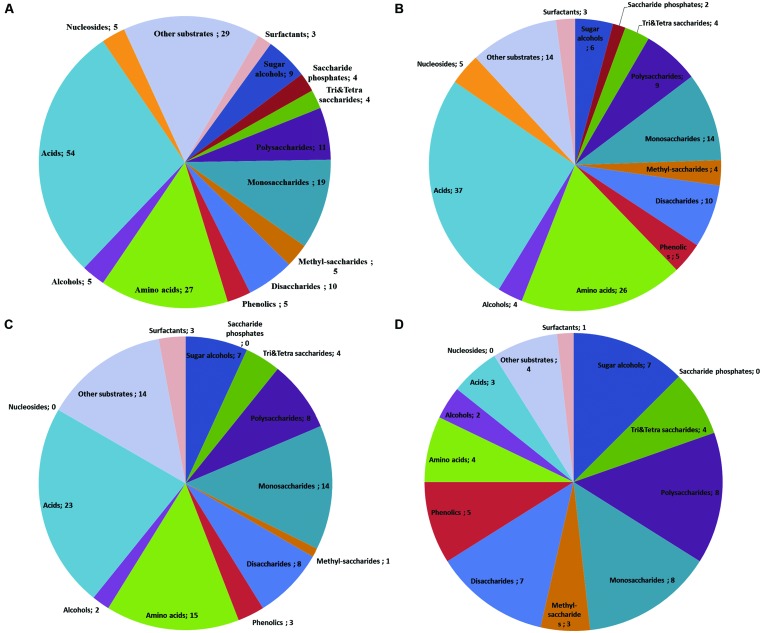
**Venn-diagrams show the utilization patterns of carbon sources (divided in chemical groups) of three fungi as indicated by the color development data on BIOLOG plates Phenotype MicroArray (PM) 1 and 2A.** All available substrates divided in chemical groups (number of individual compounds after the semicolon; **A**). Substrate utilization pattern by the endophyte *Monographella nivalis* var *neglecta* – a strong *in vitro* competitor of the pathogen, occupying the same niches in the tree and utilizing carbon sources more efficient than the pathogen **(B)**, the pathogen causing Dutch elm disease, *O. novo-ulmi* **(C)** and *Aureobasidium pullulans* **(D)**, a ubiquitous fungus with no detected competitive relation to the pathogen *in vitro* (Data modified from Blumenstein et al., 2015).

With the goal of studying the potential of endophytes in biocontrol, we used PM data to compare the nutritional preferences of a pathogen and endophytes that co-colonize the same host (**Figure 6**, data modified from Blumenstein et al., 2015). The comparison showed that all tested fungi were able to use all of the four tri-and tetrasaccharides tested (**Figure 6**). On the other hand, the endophyte *M. nivalis* was able to use a broader array of available amino acids (96%) and other acids (69%) as compared to the pathogen (56 and 49%, correspondingly) and *A. pullulans* had generally low preference for acids, utilizing 15% of the available amino acids and less than 1% of the other acids (**Figure 6**). Moreover, the endophytes were able to use all available phenolics (5 out of 5) while the pathogen could only use 60% of them (3 out of 5).

**FIGURE 7:**
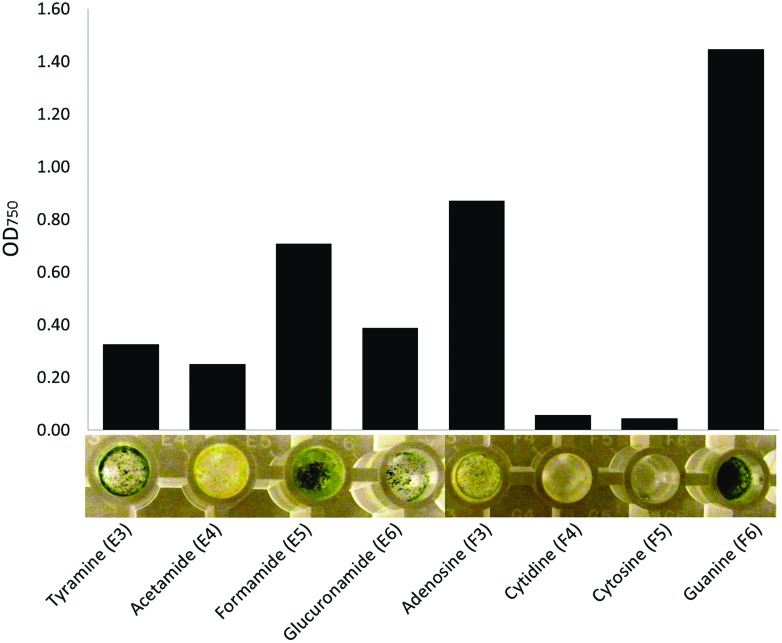
**An assortment of nitrogen sources leading to clearly visible differences in optical density (750 nm) and morphology of the fungal mass of *Trichoderma harzianum*.** Measurement at 144 h after inoculation to the plates (bars represent the mean of readings from two plates).

Phenotype MicroArray technique also allowed us to observe the effect of substrates on the morphology of the tested fungi. In particular, nitrogen sources seemed to induce varying morphological responses. For instance, only little fungal mass was produced when *T. harzianum* grew on cytidine or cytosine (**Figure 7**). Tyramine and formamide triggered production of in green fungal mass, whereas acetamide resulted in yellow, and adenosine yellow–green, fungal mass. Guanine induced formation of dense, dark green fungal mass in *T. harzianum*.

#### Procedure II – Targeted Test of Chemical Sensitivity

**FIGURE 8:**
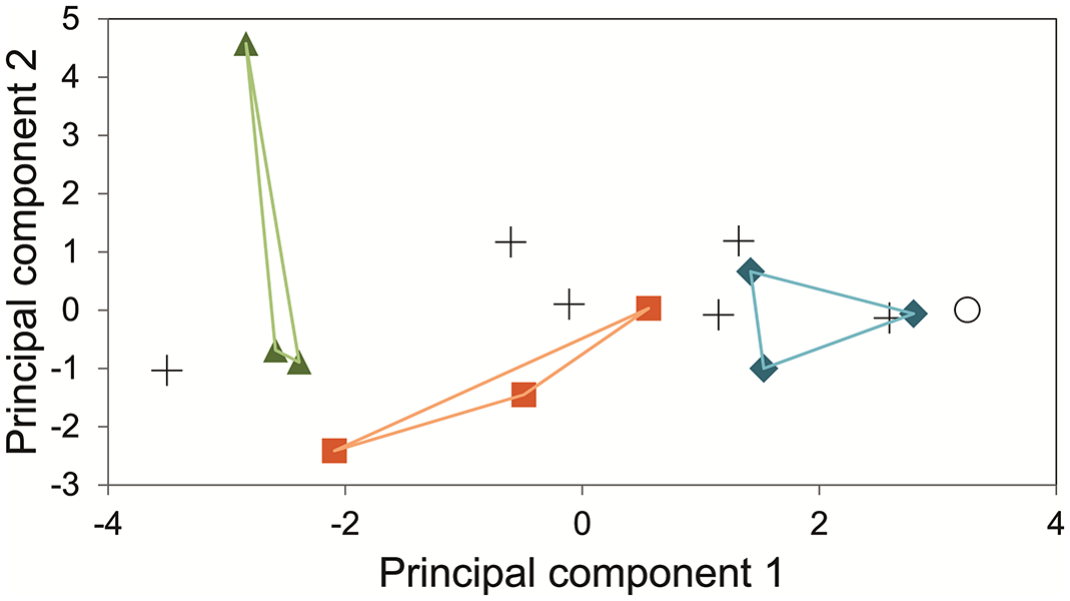
**Principal Component analysis of the cumulative growth (time point 9 dai, λ = 630 nm) of 15 fungal strains in an array of in-house PM supplemented with 11 inhibitory substances and glucose.** Green triangles indicate three strains of the family Dothioraceae. Orange squares represent members of the order Pleosporales (families Pleosporaceae and Phaeosphaeriaceae). Blue diamonds for the basidiomycetes assayed. Crosses represent a variety of ascomycetous endophytic fungi (Davidiellaceae, Lophiostomataceae, Botryosphaeriaceae, two sordariomycetes, and one *incertae sedis*). Empty circle represents the negative control without inoculum. Principal component 1 explains 42.76% and principal component 2 does 21.50%.

The measurements of the inhibitory effects of eleven substances on fifteen fungal strains (13 of them endophytes) were analyzed by means of Principal component analysis. The two main principal components collated the fungi following its phylogenetic relations (**Figure 8**).

## Discussion

### Benefits of PM Approach in Endophyte Studies

Our studies demonstrate that the PM approach is a useful tool to investigate the cellular phenotypes of forest tree endophytes at semi-high throughput rate and in a standardized manner, and to functionally interpret the taxonomic data generated by NGS. For instance, in a recent study exploring the role of endophytes in the Dutch elm disease complex, we were able to identify nutritional niche overlap as a potential mechanism of interaction between the pathogen and potential antagonist endophytes (Blumenstein et al., 2015). This information will assist selection of candidate endophytes for biocontrol purposes. Moreover, we successfully applied PM to characterize the degradation capabilities of the main fungal taxa operating during the first stages of eucalypt wood decay (Macaya-Sanz et al., 2014). In that work, a succession of the most abundant endophyte fungi present during the first 70 days of wood degradation was monitored through pyrosequencing fungal ITS1 region. The resulting operational taxonomic units (OTUs) frequencies varied in time, and certain endophyte OTUs orders were abundant at the beginning of the degradation. Furthermore, the PM analysis showed that these orders are able to effectively degrade lignin-like substances, while other OTUs prevailed at the end and were favored by presence of lignin degradation by-products. This information may be valuable for wood processing industries, but it can also add to the current scientific discourse about the role of microbes as regulators of carbon balance in forest ecosystems (Hiscox et al., 2015) and support the decision making regarding conservation of biodiversity in our forests. Thus, PM techniques are useful tools for both basic and applied research, and can be successfully applied in highly different research fields, such as plant protection, wood material research, and conservation ecology.

Based on our experiences, we conclude that the general benefits of PM approach include its great versatility that allows various research questions to be addressed in a same experiment (e.g., testing of competitive relationships between fungal strains along with gaining information about their sensitivity to individual chemical compounds), testing of a broad array of different compounds and concentrations, and a higher throughput of samples, as compared with earlier methods that have been used in studies of fungal phenotypes (Yourman et al., 2001; Atallah et al., 2011). A great advantage was also that the phenotypic responses are recorded quantitatively and stored electronically (Bochner, 2003; Bochner et al., 2010). If the technical challenges and limitations are properly acknowledged (see below), PM approach opens new experimental possibilities for tree-endophyte research.

### Technical Challenges with PM Method

One of the fundamental challenges when working with fungi in the PM procedure is to prepare a representative, homogenous and viable inoculum. Part of this challenge is because the external growth conditions can strongly modulate the quality and quantity of inoculum. Fungi are known to show great phenotypic plasticity in their responses to their immediate growth environment (see, e.g., Rohlfs, 2015, and references therein). In accordance with this expectation, also our results from the nitrogen tests (Procedure I, **Figure 7**) witness how strongly the substrate can affect fungal morphology, which in turn is a product of the fungal metabolism. Moreover, we found a marked loss of vitality during long term storage, possibly because the month of storage promoted the strains to enter latent stages, from which the fungi could not completely recover on the MS medium. Thus, the conditions before preparation of inoculum may influence the responses of fungal cells on PM plates through metabolic carry-over effects. As a first step when using PM approach, whether it is in-house or pre-configured PM plates, we thus recommend careful standardization and documentation of the pre-inoculum growth conditions for the fungi, to ensure the repeatability of analyses.

Standardization of growth conditions may, however, also add bias to the analysis, given the strain-specific preferences for optimal growth. Indeed, in both procedures (I and II), we found evidence for strain-specificity in fungal responses. For instance, the above mentioned conditioning effects of pre-inoculum preparation growth environment could be highly genotype-specific because the nutritional niches of the strains differ (Blumenstein et al., 2015). Therefore, the possible carry-over effect of culturing conditions may be a factor that needs to be taken into consideration in particular when comparing interspecific differences. In our studies, we pre-cultured all fungi on MEA and at 26°C even though their individual growth preferences may differ. Ideally, the species-specific nutrient utilization patterns detected through PM analysis should be validated using inoculum collected from colonies of the same isolates that have been cultured under a well-defined set of pre-conditions that cover the realistic regimes in physicochemical environment (light, temperature, pH, etc.). Standardization of the pre-inoculum preparation conditions could also be done on strain-specific basis, ensuring the strain-specific maximum growth rate. An interesting possibility with PM could also be to study the possible carryover effects with the goal of gaining a better understanding of how we could better gear the fungal phenotypes for different industrial or pharmaceutical purposes.

Intriguingly, we also observed (Procedure II) how complex strain-specific interactions with the chemical environment were expressed as unexpected color changes in the wells (**Figure 4**). The orange color is characteristic for oxidation of phenols (Holderbaum et al., 2010) and could thus be indication of fungal, extracellular phenolase activity. Another possibility is that the chemical environment induced the fungi to produce colored substances (Velmurugan et al., 2010). Thus, in order to avoid confounding effects of such within-well processes, it is necessary to carefully plan the positive and negative controls in each in-house array. Moreover, new research is needed to better understand the metabolic processes in fungal cells as they grow in the wells.

The second essential step in use of PM approach is to establish an inoculum preparation routine that ensures good viability and accurate and repeatable quantification of inoculum. Our method for preparing a homogenous inoculum (Procedure I) resulted in dense emergence of hyphae on the Petri dishes within 2–4 days, indicating that the process did not negatively affect the viability of the cells. According to the manufacturer’s protocol, the density of the IF should be set to 62% transmittance, conveniently measured from inoculum fluid tubes with the original Biolog turbidimeter. Bochner (2009) states that technically one cell per well would be adequate, but recommends 100 cells per 100 μL for the inoculum. For bacterial cells, a concentration of approximately 10^6^ cells/mL is a common standard (e.g., Bourne et al., 2012). For fungi, Atanasova and Druzhinina (2010) recommend an adjusted cell density ranging from 1.25 × 10^5^ to 5 × 10^5^ spores/mL, depending on the tested fungus to guarantee repeatable OD measurements.

Insam et al. (1996), however, suggest that instead of total counts, viable counts for microorganisms should be used, or the biomass should be standardized. In our Procedure II, we used biomass standardization. This method is practical in particular for those endophytes that do not readily sporulate in cultures. However, the proportion of metabolically non-active biomass (e.g., non-active hyphal segments) should ideally be controlled as a part of the protocol, by microscopic examination or by determining the CFU of the inoculum. For adequate repeatability of the results, efforts should be made to guarantee that the inoculum prepared from different isolates of a same species contain a comparable CFU/mL and that each well contains at least 100 cells per well (Bochner, 2009).

The third crucial step in PM analyses, in particular when working without the OMNILOG instrumentation and software, is to decide the time points of interest for data collection. There is a temporal dynamic in the substrate use by the cells, which is a fundamental to the evaluation of cell phenotypes. The reaction in the wells is often characterized by a lag-period that can last up to 2–4 days. After that the reaction develops and finally tends to saturate. In the case of the fungi that we have studied the saturation often started after 96 h from the start of the inoculation period. Hierarchical cluster analysis proved to be a useful tool for determination of the appropriate time for downstream analyses of the substrate use by the fungi (Blumenstein et al., 2015). In cases where such clear cluster separation of the replicate measurements does not exist, the readings that represent the highest degree of strain separation could be chosen for further analyses. In our Procedure I studies, we found that the readings for the studied fungi (a pathogen and three fungal endophytes) were most reliable between 168 and 240 h after incubation. We also found that viable cells could be recovered from the wells even after 360 h on the plates. An intriguing option to further utilize PM technique would be to extract the fungal biomass in the wells in the end of an incubation period and study, e.g., using chromatography and molecular approaches, how growth on a single substrate might affect the capacity of fungal cells to accumulate specific chemicals, or express certain genes.

For the in-house configured PMs, we also identify a fourth crucial step: Our Procedure II studies demonstrated that the repeatability and reliability of the in-house arrays can only be ensured through a careful design that acknowledges the specific characters of the studied chemicals. For instance, the volatility of a compound dispensed in a certain well could affect fungal growth in surrounding wells (e.g., observed in adjacent wells to inhibitory, volatile *o*-cresol; **Figure 3**). Thus, when analyzing the chemical sensitivity of volatile substances, a preferable approach seems to be dispensing the same concentration of the volatile substance in question in all the wells of the microplate in order to avoid unintentional cross-well effects.

### Challenges in Interpretation of the Biolog Data

The PM technique is a powerful tool to estimate the relative speed of substrate use for particular fungi. However, the interpretation of the between-species difference in the speed of substrate use is not straightforward. A change is absorbance values can be interpreted as a proxy of metabolic activity, but it is risky to propose narrower views. The change of color could be produced by a shift in the tetrazolium dye due to respiration, by an increase of turbidity through fungal body proliferation or even by change of the medium color after production of metabolites by the fungi. Such mélange of processes makes it challenging to contrast different organisms in a fine scale. The inconsistency of results in the repeated assays where six strains were tested on seven carbohydrates (**Table 1**) may reflect such blended processes that interplayed with the inherent variations in the enzymatic activities of the fungi (e.g., van den Brink and de Vries, 2011). Thus, until refinement, the procedure is best suited for studies on general metabolic trends.

Despite the uncertainty, the proxy of metabolic activity can be valuable additional information, e.g., in studies addressing the potential endophyte-based applications (see Benefits of PM Approach in Endophyte Studies). In our recent study (Protocol I; Blumenstein et al., 2015), we used pre-configured PMs to deepen our understanding of the mechanisms of antagonism by potential biocontrol endophytes, identified on basis of field correlations and laboratory tests. In this case, the PM data allowed us to nuance the emerging picture of the potential of endophytes in biocontrol: we could conclude that the antagonistic effect of an endophyte against a pathogen may be due to several, simultaneous or parallel mechanisms (chemical antagonism and competition for nutrients). PM analysis also provided information about the possible ecological strategies of the fungi. In particular, we found that the ubiquitous *A. pullulans* is a slow substrate user with a relatively high tolerance to potentially antifungal compounds such as phenolics (**Figure 6**), whereas the biologically more specialized species *M. nivalis* var. *neglecta* and *P. cava*, were capable of faster catabolism of certain substrates, e.g., monosaccharides, polysaccharides and acids, but were less tolerant to phenolics as compared to *A. pullulans*. These results indicate that while *M. nivalis* var. *neglecta* and *P. cava* may be superior competitors in sugar- and acid-rich environments, *A. pullulans* may have an ecological strategy that permits it to remain active even on nutritionally more reluctant substrates that might cause other fungi to starve or become intoxicated. Given that *M. nivalis* var. *neglecta* and *P. cava* have been identified as potent inhibitors of a vascular pathogen of the host species, elms (Blumenstein et al., 2015), this information might help to set chemical quality targets for the elm trees that are most receptive for biocontrol through these endophytes. Similarly, the protocol II study showed that the phylogeny could be considered as a proxy of the fungal response to plant secondary metabolite. This kind of results, in combination with NGS analyses, can provide novel information about the mechanisms behind the structure and functions of endophyte communities in trees.

The obviously artificial growth environment in PM plate wells, and the distant resemblance of preconfigured plates with the substrate availability under natural conditions, may obviously obscure the interpretations. We only see the response to one isolated substrate in each well on a PM plate. In nature, however, fungal cells inside the plant tissues will exist in a chemical environment that is likely to constantly and gradually change. This dynamicity will be caused both by the metabolism of the plant and by the endophytic inhabitants, and the composition of the substrate, as well as the fungal community, will continuously be altered. Similar successions have been studied for macro-organisms including fungi and insects in decaying leaf litter (Hättenschwiler et al., 2005) or the succession of saprotrophic organisms in dead woods of forests (Bader et al., 1995, Similä et al., 2003). As one of the functions of endophytic fungi is likely that of a decomposer (Schulz and Boyle, 2005), the conversion from one substrate to another by the help of a fungi will likely determine the succession of endophyte function in a plant both during its life as well as during its after life.

In natural conditions, variations in species-specific infection mode and presence of specific endophytes are also determining the use of substrates by a fungus, which, in turn, will affect the succession of endophytes (Heilmann-Clausen and Boddy, 2005). Thus, the order in which endophytic fungi enter the host plant may also determine the activity and importance of subsequent endophytes in that plant. It further suggests that there is plasticity in how the fungi may make use of the plant, in the sense that some individual species may play different roles depending on when they enter the scene. A certain fungal endophyte may therefore be plastic in the ecological role they play, which may further complicate the interpretations of the biological significance of the phenotypic responses detected in PM plates.

## Conclusion

The technical challenges of the PM method are multifaceted and the interpretation of PM data is not straightforward. Thus, ideally, extensive validation through carefully standardized pre-conditions for the fungal growth and careful replication and control well strategies are needed for successful PM analyses, whether the studies use preconfigured or in-house designed PM plates. However, it is evident that the PM technique may significantly help to bridge the genotype–phenotype gaps for the culturable fraction of endophytic fungi. Despite the above-mentioned challenges, PM analyses can provide unique knowledge about functional properties of individual strains and species, and thereby contribute to the knowledge pool that is needed for a more comprehensive understanding of the associations between the NGS-profiles and functional fungal biodiversity.

## Conflict of Interest Statement

The authors declare that the research was conducted in the absence of any commercial or financial relationships that could be construed as a potential conflict of interest.
